# Supplementary material for: Investigation of Broadband Optical Nonlinear Absorption and Transient Dynamics in Orange IV Containing Azobenzene
Source: Molecules. 2023 Jun 10;28(12):4692. doi: 10.3390/molecules28124692 (PMC10303707; doi:10.3390/molecules28124692)
Supplement: Supplementary file 1 [file molecules-28-04692-s001.zip › molecules-2363327-supplementary.pdf]

Supporting Information

Investigation of broadband optical nonlinear absorption and transient dynamics in Orange IV containing azobenzene

Quanhua Wu [1], Rui Ruan [1], Xingxing Li [1], Yujie Zhao [1], Yang Li [1], Yu Fang [1], Yongqiang Chen [1], Quanying Wu [1], Yinglin Song [2,3,*] and Xingzhi Wu [1,*]

1 Jiangsu Key Laboratory of Micro and Nano Heat Fluid Flow Technology and Energy Application, School of Physical Science and Technology, Suzhou University of Science and Technology, Suzhou 215009, China; wuquanhua1998@163.com (Q.W.); 17774003013@163.com (R.R.); lixing17339649786@163.com (X.L.); 18361793147@163.com (Y.Z.); liyang@usts.edu.cn (Y.L.); yufang@usts.edu.cn (Y.F.); yqchen@usts.edu.cn (Y.C.); wqycyh@mail.usts.edu.cn (Q.W.)

2 Department of Physics, Soochow University, Suzhou 215123, China

3 Department of Physics, Harbin Institute of Technology, Harbin 150001, China

* Correspondence: ylsong@hit.edu.cn (Y.S.); wuxingzhi@usts.edu.cn (X.W.)

Closed Aperture Z-scan results of solvent DMSO and Orange IV solution at 532 nm. The linear transmittance is 0.35, and the incident peak power density is 21.4 GW/cm^2

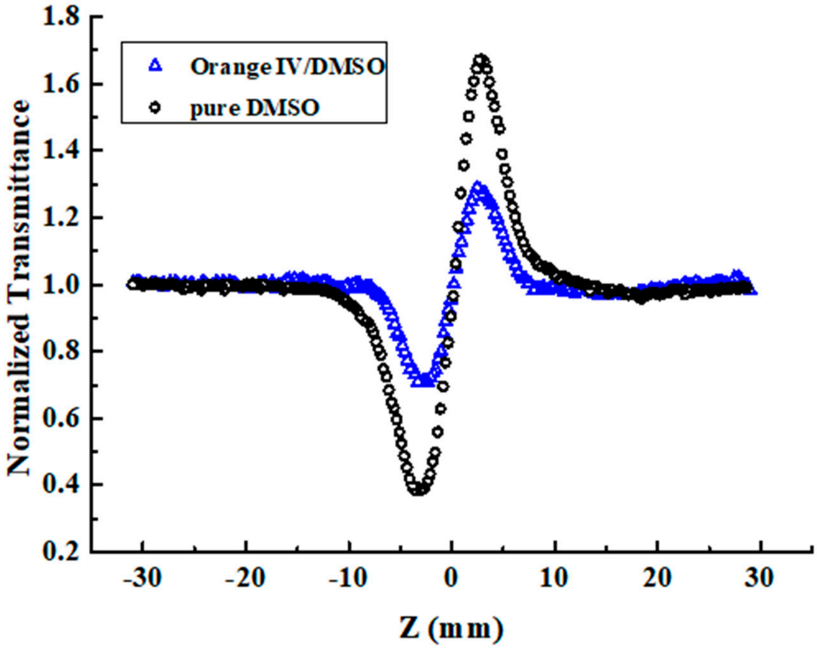

Figure S1. Closed Aperture Z-scan results of solvent DMSO and Orange IV solution at 532 nm.

The molecules studied in this paper were purchased from chemical reagent companies. Its ^{1}H NMR

spectrum is as follows:

^{1}H NMR (400 MHz, DMSO-d6) δ 8.89 (s, 1H), 7.82 (d, J = 8.9 Hz, 2H), 7.74 (s, 4H), 7.37 – 7.29 (m, 2H), 7.22 (d, J = 7.3 Hz, 2H), 7.17 (d, J = 9.0 Hz, 2H), 6.99 (t, J = 7.3 Hz, 1H).

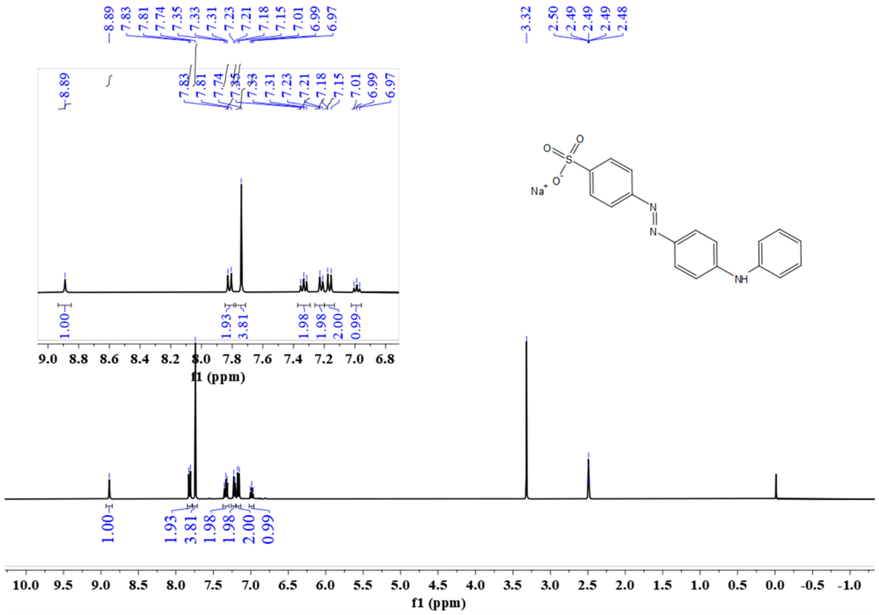

Figure S2. ^{1}H NMR spectrum of Orange IV in DMSO.
